# Patient safety incidences: Perspectives of South African audiologists

**DOI:** 10.4102/safp.v67i1.6134

**Published:** 2025-05-19

**Authors:** Suvishka Barath, Ntandoyenkosi L. Msomi, Andrew J. Ross

**Affiliations:** 1 Department of Family Medicine, Faculty of Health Science, University of KwaZulu-Natal, Durban, South Africa

**Keywords:** patient safety incidents, audiology, qualitative research, health care training, South Africa

## Abstract

**Background:**

Patient safety incidents (PSIs) in audiology have received limited attention compared to other health care professions in South Africa, despite their potential to significantly impact patient well-being. This study explores audiologists’ experiences of PSIs and the factors contributing to their emergence.

**Methods:**

A qualitative, contextual, descriptive exploratory research design was employed. Individual semi-structured online interviews were conducted with eight audiologists working in South Africa. The data were analysed using Braun and Clarke’s reflexive thematic analysis.

**Results:**

Participants highlighted that PSIs in audiology are often underestimated, yet they can profoundly affect patients’ quality of life. Inadequate university training on PSIs was identified as a key contributing factor. Technological advancements, such as over-the-counter hearing aids and automated assessments, were viewed as potential risks without proper professional oversight. Organisational support varied, with clearer protocols observed in the public sector than in private practice. In addition, staff shortages and high workloads increased the likelihood of PSIs by compromising patient care. To mitigate these risks, participants recommended enhanced training, stricter regulation of hearing technologies and improved patient education.

**Conclusion:**

This study underscores the need for greater recognition and proactive management of PSIs in audiology. Addressing training gaps, strengthening organisational support and implementing regulatory measures for emerging technologies are essential to improving patient safety outcomes.

**Contribution:**

This study expands the understanding of PSIs in audiology and offers insights that can inform curriculum reform and professional development initiatives in South Africa.

## Introduction

Patient safety is a cornerstone of health care that is aimed at minimising harm during the delivery of services.^1^ The World Health Organization (WHO) emphasises the need to reduce patient safety incidents (PSIs), these being unintended or unexpected events that negatively affect patients, including diagnosis, treatment and communication errors.^2^ Patient safety is particularly relevant in audiology, as the management of hearing and vestibular disorders directly influences patient well-being and recovery.^3^ Approximately 1.5 billion people globally live with hearing loss, profoundly impacting their daily functioning and quality of life (QoL).^4^ Of these individuals, 80% reside in low-income and middle-income countries (LMICs), such as South Africa, where health care systems may struggle to meet their needs.^5,6^ Disabling hearing loss is more prevalent in LMICs, such as sub-Saharan Africa (SSA) and South East Asia^7^ contributing to communication barriers, social isolation, and limited educational and occupational opportunities.^8^ These issues underscore the need for effective audiological care to prevent diagnostic errors, treatment delays and other safety concerns.

Audiologists are uniquely trained to diagnose and treat hearing disorders, making their role essential in addressing issues related to hearing loss.^3,8^ Audiologists also possess specialised expertise in evaluating and managing vestibular disorders, making them integral to the comprehensive delivery of auditory and balance health care.^3^ Given hearing loss’s complex nature and associated conditions, precise diagnosis and effective treatment are essential for ensuring patient safety and improving outcomes.^3^ This highlights the need for enhanced training and awareness among audiologists to mitigate the risks of PSIs within this patient population.

Despite the importance of patient safety, research focussing specifically on PSIs within audiology remains limited, particularly in South Africa, where disparities in health care access and resource distribution may exacerbate safety risks.^5^ Potential PSIs in audiology may include misdiagnosis of auditory conditions, incorrect hearing aid fittings, inadequate counselling on assistive devices and improper cerumen management.^3^ In addition, vestibular disorders increase the risk of falls,^9^ a major reportable safety issue in clinical and hospital settings. Research has shown vestibular dysfunction to have higher morbidity and mortality rates because of falls, reinforcing the necessity for enhanced patient safety measures within audiology.^10^ These risks can lead to significant harm, including delays in appropriate interventions and compromised patient outcomes, emphasising the necessity for heightened vigilance in audiological practice. Effective communication, accurate assessments and well-coordinated care plans are essential for reducing the likelihood of PSIs, especially for patients living with disabilities because of hearing loss.

Globally, incident reporting is recognised as an essential tool for improving patient safety across medical disciplines.^11^ The WHO’s *Global Patient Safety Action Plan 2021–2030* has laid the foundation for improving safety protocols worldwide, with an emphasis on reducing preventable harm across health care systems.^11^ Furthermore, the Institute of Medicine’s report *To err is human* highlighted the critical need for health care systems to adopt safety measures and incident reporting.^12^ Similarly, frameworks from organisations such as the National Health Service (NHS) in the United Kingdom (UK) and the Australian Commission on Safety and Quality in Health Care have driven patient safety improvements globally.^13,14^ These frameworks have significantly contributed to reducing PSIs in high-income countries. However, in LMICs such as South Africa, health care challenges such as resource constraints and under-reporting of incidents hinder the effective implementation of such policies, leaving gaps in patient safety and quality of care.

The National Department of Health (NDoH) introduced the *Patient Safety Incident Reporting and Learning Policy* as a guide for the standardised management of patient incidents for the South African health care sector.^15^ However, there has been no clear evidence that the introduction of the guideline has resulted in the reduction of PSIs.^16^ Furthermore, its implementation in audiology remains inadequate. Understanding audiologists’ experiences with PSIs, including barriers to reporting and learning from incidents, is essential for developing effective patient safety frameworks within the profession. Although patient safety is increasingly emphasised in health care, limited research has explored audiologists’ experiences and insights regarding PSIs. This study aimed to explore South African audiologists’ perceptions of PSIs, their experiences with incident reporting and the challenges they encounter in ensuring patient safety in clinical practice.

## Research methods and design

### Study design

This study employed a contextual, descriptive exploratory research design to explore the perspectives of audiologists on PSIs. This approach was selected to facilitate an in-depth exploration of the factors shaping participants’ experiences, providing nuanced insights into their engagement with PSIs within their professional practice.

### Setting

The study was conducted in South Africa and included audiologists who are currently practising in various clinical, industrial and educational settings. The inclusion of diverse practice settings contributed to a broad representation of perspectives on PSIs in audiology.

### Study population and sampling strategy

A criterion-based purposive sampling technique was utilised to ensure a diverse representation across different clinical settings and geographical regions. Recruitment was initiated by circulating a study poster in closed WhatsApp groups comprising South African audiologists, followed by snowball sampling, wherein referrals were requested from initial participants. The use of WhatsApp groups and snowball sampling was chosen for recruitment because of the widespread use of WhatsApp among South African audiologists for professional communication and networking. These closed groups provided a direct and efficient means of reaching audiologists from various practice settings across the country. Inclusion criteria specified that participants be registered as independent audiologists with the Health Professions Council of South Africa (HPCSA), have at least 1 year of independent clinical experience and be actively practising in South Africa. Audiologists dually qualified in speech therapy and audiology were excluded to prevent data confounding.

The final sample comprised eight participants. This sample size aligns with qualitative research principles, where depth of insight is prioritised over statistical representativeness.^17^ Data saturation was achieved when no new themes emerged from additional interviews, justifying the sufficiency of the sample size.

### Data collection

Participants who expressed interest received an information sheet and consent form before interview appointments were scheduled. Semi-structured interviews were conducted via WhatsApp voice calls to enhance accessibility and flexibility. An interview guide (Appendix 1), developed from existing literature, structured the discussions through predetermined and probing questions, ensuring a comprehensive exploration of participants’ experiences. Interviews lasted between 30 and 50 min, were recorded with participant consent and transcribed verbatim for analysis.

A pilot study was conducted with two audiologists before data collection commenced. This process refined the interview questions, ensuring clarity, relevance and logical sequencing. The pilot study also provided insights into the estimated duration and resource requirements for the main study. Minor modifications were made to question phrasing to enhance clarity based on participant feedback.

### Data analysis

The study utilised a reflexive thematic analysis (RTA) approach, allowing themes to emerge from the data while considering the researcher’s reflexive role in the interpretative process.^18^ In keeping with RTA, an inductive approach was utilised, with three principal approaches, as developed by Braun and Clarke,^18^ being used to analyse the data: (1) coding reliability thematic analysis; (2) codebook approaches to thematic analysis; and (3) the reflexive approach to thematic analysis.^19,20^ The iterative nature of the RTA ensured that the emerging themes were contextually grounded in the participants’ responses. The analysis followed a structured process:

- Data familiarisation: Audio-recorded interviews were transcribed verbatim to ensure the accuracy of analysis. The researchers engaged in repeated reading of the transcripts to enhance familiarity with the data.
- Systematic coding: Transcripts were uploaded into NVivo 14 (Lumivero, Denver, Colorado, USA), a qualitative data analysis software, to facilitate systematic coding. Codes were generated inductively, capturing meaningful patterns and ensuring a data-driven approach.
- Theme development: Codes were grouped into potential themes based on conceptual similarities, ensuring that emerging themes reflected participants’ perspectives.
- Iterative refinement: Themes were reviewed, refined and defined through an iterative process to ensure coherence and contextual grounding. Constant comparison within and across data sets was conducted to enhance the data analysis process.
- Reflexivity and rigour: The researcher’s role in interpretation was continuously acknowledged through reflexive journaling ensuring transparency and minimising potential biases.
- Credibility measures: To enhance analytical rigour, an independent coder was engaged to review the coding process and thematic structure. Differences in interpretation were discussed and consensus was reached, further strengthening the trustworthiness of the findings.

### Trustworthiness

To enhance the credibility and rigour of the study, strategies aligned with Lincoln and Guba’s^21^ model of trustworthiness were implemented:

- Credibility: Reinforced through prolonged engagement with the data, reflexive journaling to minimise researcher bias and the involvement of an independent coder to validate the coding process and thematic structure
- Transferability: Ensured through rich, thick descriptions of the participant experiences, allowing for applicability to similar settings
- Dependability: Maintained via an audit trail documenting all research decisions, coding processes and theme development to allow for transparency
- Confirmability: Achieved through ongoing reflexive practice and peer debriefing with co-researchers to validate interpretations and reduce subjective bias.

### Ethical considerations

Ethical approval was obtained from the University of KwaZulu-Natal Humanities and Social Sciences Research Ethics Committee (HSSREC/00007277/2024). Participants’ anonymity and confidentiality were ensured by omitting identifying details from transcripts and reports. All data were securely stored on a password-protected device, accessible only to the researchers of this study.

## Results

### Characteristics of the participants

The study included eight audiologists from five provinces in South Africa, all within a similar age range (24 years – 26 years). There was an equal representation of males (*n* = 4) and females (*n* = 4), as well as between those working in private practice (*n* = 4) and public practice (*n* = 4). A summary of participants’ demographics is presented in Table 1.

**TABLE 1:** Participants’ demographics.

Participant no.	Age (years)	Gender	Province	Years of experience	Current practice facility
1	24	Male	Gauteng	3	Public hospital
2	24	Male	North-West	3	Private occupational setting
3	25	Female	Eastern Cape	3	Private practice
4	25	Male	KwaZulu-Natal	4	Public hospital
5	24	Female	KwaZulu-Natal	3	Private practice
6	26	Female	KwaZulu-Natal	4	Private practice
7	24	Male	Western Cape	3	Public hospital
8	24	Female	Gauteng	3	Public education facility

No., number.

Six themes emerged from the study with 12 sub-themes being identified. Table 2 provides a summary of the identified themes and sub-themes.

**TABLE 2:** Themes and sub-themes.

Themes	Sub-themes
1. Perceptions and impacts of PSIs	1.1. Underestimating audiology-related risks 1.2. Long-term impact on patient well-being
2. Contributing factors to PSIs	2.1. Inadequate university training 2.2. Clinical challenges in practice
3. Technology and the risk of bypassing professional oversight	3.1. Risks of self-fitting over-the-counter hearing aids 3.2. Limitations of automated hearing tests
4. Organisational systems for PSI	4.1. Inconsistent reporting systems 4.2. Institutional support in public settings
5. A lack of staff and high workloads	5.1. Impact on service quality 5.2. Delays in patient care
6. Recommendations for minimising PSIs	6.1. Enhancing training and education 6.2. Regulation of technology and professional oversight

PSIs, patient safety incidents.

#### Theme 1: Perceptions and impacts of patient safety incidents

Participants emphasised that PSIs in audiology are often underestimated, despite their significant impact on patients’ QoL. They highlighted that although their field is not traditionally associated with high-risk procedures, even minor errors in diagnosis or intervention could affect a patient’s ability to communicate or lead to significant balance and coordination difficulties ultimately having an impact on their social and emotional well-being. Two sub-themes emerged for this theme, these being understanding audiology-related risks and consequences of QoL.

**Sub-theme 1.1: Underestimating audiology-related risks:** Participants expressed that audiology is often perceived as a low-risk field, with many health care providers failing to recognise the potential for significant patient harm because of errors in diagnosis or treatment. Despite the nature of audiology not involving life-threatening procedures, participants stressed that even minor mistakes can have serious consequences for patients:

‘People think PSIs are only for doctors… but if I do not diagnose hearing loss correctly, or if I fit a hearing aid incorrectly, the patient’s communication is going to be seriously affected.’ (Participant 1, 24-years-old, male)

‘What we do affects how people engage with the world. If something goes wrong during an audiological procedure, it can have long-term effects on a patient’s ability to function in society.’ (Participant 6, 26-years-old, female)

**Sub-theme 1.2: Long-term impact on patient well-being:** While audiology may not involve life-threatening situations, participants highlighted that errors in the field can significantly affect patients’ ability to engage in everyday activities, leading to negative emotional and social outcomes. Participants emphasised that mistakes in audiology can result in long-lasting issues, such as social withdrawal or even depression, by impacting patients’ communication or balance:

‘What we do directly affects communication and balance. If I fit a hearing aid poorly or do not manage hearing loss correctly, the patient will withdraw from social situations, and it could even lead to depression.’ (Participant 3, 25-years-old, female)

‘PSIs in audiology are about quality of life. If a patient is over-amplified, it can damage their hearing further, which is not as visible as a surgery mistake, but is still harmful.’ (Participant 4, 25-years-old, male)

‘Some health care providers do not see the risks in audiology, but we are dealing with patients’ ability to hear, function and communicate. It is about their quality of life, not just clinical outcomes.’ (Participant 2, 24-years-old, male)

#### Theme 2: Contributing factors to patient safety incidents

Participants highlighted a lack of formal training on managing PSIs as a significant contributing factor to the incidents they may encounter. The two sub-themes that emerged related to inadequate university training and clinical challenges in practice left them unprepared to handle real-world incidents.

**Sub-theme 2.1: Inadequate university training:** Participants reported that their university education needed to prepare them for PSIs, as the topic had not been raised during their undergraduate training:

‘We were not taught about PSIs at all in university. I did not even know what it meant until I started working.’ (Participant 2, 24-years-old, male)

‘University focused on the technical side, but there was little on how to handle incidents. Now in practice, I am facing real-world situations that we were never taught to manage.’ (Participant 5, 24-years-old, female)

‘I have heard about incidents, like when a patient collapses in the booth or falls during vestibular testing, and we do not know the protocols. We were not taught about these incidents at university.’ (Participant 2, 24-years-old, male)

**Sub-theme 2.2: Clinical challenges in practice:** Participants described several clinical challenges, particularly in ear mould impressions and wax removal, where errors could lead to harm:

‘You are dealing with delicate areas like the ear canal. A small mistake during wax removal could perforate the eardrum.’ (Participant 8, 24-years-old, female)

‘One time, a patient started bleeding during the session. We had no idea how to handle it, and it was frustrating that there was not more support.’ (Participant 6, 26-years-old, female)

#### Theme 3: Technology and the risk of bypassing professional oversight

Participants highlighted concerns regarding patients self-managing their hearing care without professional input, facilitated by advancements such as over-the-counter (OTC) hearing aids and automated hearing assessments. While these technologies improve accessibility, they also pose risks when patients use them without proper guidance, leading to mismanagement, potential hearing damage and delayed intervention.

**Sub-theme 3.1: Risks of self-fitting over-the-counter hearing aids:** Participants raised concerns that OTC hearing aids allow patients to bypass professional assessment, increasing the risk of improper fitting, inadequate amplification or even hearing damage. Without audiological oversight, patients may unknowingly select inappropriate devices or misuse them, leading to complications that require professional intervention:

‘With OTC hearing aids, patients do not always know what they are doing. They could be over-amplifying their hearing, causing more damage.’ (Participant 3, 25-years-old, female)

‘With over-the-counter hearing aids, patients are fitting these devices themselves without professional oversight. That is risky, because if the device is too powerful or not powerful enough, it could damage their hearing or worsen the problem.’ (Participant 2, 24-years-old, male)

‘Technology is great, but it also means less control over the process. We are seeing patients come in with hearing aids they bought online, and they have been misusing them, which creates safety issues.’ (Participant 4, 25-years-old, male)

‘I have had patients come in with OTC devices that were completely wrong for them, and now I have to undo the damage caused.’ (Participant 4, 25-years-old, male)

**Sub-theme 3.2: Limitations of automated hearing tests:** Participants expressed concern that automated hearing tests, including self-administered online screenings, lack the nuanced interpretation that trained audiologists provide. While these tools can facilitate early identification of hearing loss, they may also lead to misdiagnosis, inappropriate hearing aid selection or delayed professional assessment:

‘Automated hearing tests are becoming common, but they can miss important details. A machine cannot pick up on certain subtle signs that an audiologist would.’ (Participant 6, 26-years-old, female)

#### Theme 4: Organisational systems for patient safety incidents

The participants’ experiences with organisational support for handling PSIs varied. Those working in public sector hospitals reported inconsistent reporting protocols, while those in private practice described a lack of clear procedures.

**Sub-theme 4.1: Inconsistent reporting systems:** Audiologists working in private practice expressed frustration over the absence of formal reporting systems for PSIs, leaving them needing guidance on managing incidents:

‘We do not have a formal system for reporting PSIs. If something goes wrong, we are just expected to figure it out.’ (Participant 3, 25-years-old, female)

‘In private practice, it is often up to you to manage incidents, which can be very stressful, especially when you do not have support.’ (Participant 5, 24-years-old, female)

**Sub-theme 4.2: Institutional support in public settings:** In contrast, those working in public sector hospitals described the availability of structured protocols, including reporting channels and institutional support for handling PSIs:

‘There are protocols in place, so we know exactly what to do if a PSI occurs, which takes much pressure off.’ (Participant 4, 25-years-old, male)

#### Theme 5: A lack of staff and high workloads

An important theme that emerged was the impact of staff shortages and high patient volumes on patient safety; the lack of staff being a critical factor leading to PSIs, particularly in public sector hospitals, where audiologists often have overwhelming caseloads.

**Sub-theme 5.1: Impact on service quality:** Many participants expressed concerns that being understaffed compromised the quality of care they could provide and that they felt pressured to rush through assessments, which increased the likelihood of errors:

‘There is not enough staff, so we are constantly rushing to see patients. Sometimes I feel like I am missing things because we just do not have enough time.’ (Participant 7, 24-years-old, male)

**Sub-theme 5.2: Delays in patient care:** Staff shortages also resulted in significant delays in care, which could lead to deterioration in patients’ conditions or delayed interventions:

‘Patients often wait months for follow-up appointments because we do not have enough staff. By the time they come back, their hearing has gotten worse.’ (Participant 1, 24-years-old, male)

‘We are so short-staffed that urgent cases sometimes do not get seen right away, and that can make their condition harder to treat.’ (Participant 1, 24-years-old, male)

#### Theme 6: Recommendations for minimising patient safety incidents

Based on their perceptions and experiences, participants recommended several ways to minimise PSIs in audiology, focussing on improving training and education and regulating new technologies.

**Sub-theme 6.1: Enhancing training and education:** Participants emphasised the need for more comprehensive training on PSIs during university education and professional practice:

‘We need more training on PSIs at university, but also in the workplace. Continuous training would make a huge difference.’ (Participant 6, 26-years-old, female)

‘If we had more refresher courses on patient safety, I think we would be much better prepared to handle these situations.’ (Participant 7, 24-years-old, male)

**Sub-theme 6.2: Regulation of technology and professional oversight:** Participants called for tighter regulation of new technologies, particularly OTC hearing aids, to ensure that patients did not put themselves at risk of causing avoidable injuries:

‘There should be stricter regulations for OTC hearing aids. Patients need to be educated on how to use them properly, or we will see more incidents.’ (Participant 4, 25-years-old, male)

‘The technology is moving fast, but we need to keep up with safety regulations, so patients are not left on their own.’ (Participant 5, 24-years-old, female)

## Discussion

This study explored South African audiologists’ perceptions of PSIs, identifying key contributing factors and recommendations for improvement. To the authors’ knowledge, this is one of the first studies to explore PSIs from the perspective of audiologists in South Africa. The findings highlight critical yet often overlooked risks in audiology, emphasising the need to prioritise patient safety.

A significant concern identified was the underestimation of audiology-related risks, with participants reporting that PSIs in their field are not perceived as critical by other health care professionals. This aligns with the broader challenges of underfunding and undervaluation of audiology services in South Africa, contributing to a lack of awareness regarding the long-term consequences of untreated or mismanaged hearing loss.^22^ While audiology may not present immediate life-threatening risks, errors such as misdiagnosing hearing loss or incorrect hearing aid fittings and management of vestibular disorders can severely impact a patient’s QoL, communication abilities and social integration. These findings align with literature emphasising that untreated hearing impairment increases listening effort, impacting communication, language acquisition and speech recognition.^23^ This underscores the need for a paradigm shift in recognising the serious nature of PSIs in audiology. In South Africa, untreated hearing loss is already a public health concern, particularly in rural communities that have limited access to audiological care.^8,22^ The cumulative effect of undiagnosed or mismanaged hearing loss by audiologists in these populations amplifies the potential harm of PSIs. This emphasises the need to place patient safety at the forefront of audiological care across all settings, from urban centres to remote regions, where audiologists may work in isolation or without adequate support structures.

As identified in this study, a significant contributing factor to PSIs was the need for more comprehensive training in university programmes and clinical settings. The gaps in the audiology curriculum relating to patient safety highlight a missed opportunity to prepare audiologists for real-world challenges. The findings of this study align with an Ethiopian study that reported poor documentation practices among health professionals and showed that insufficient training was associated with sub-optimal adverse event reporting.^24^ Furthermore, the lack of reporting mechanisms and poor documentation can significantly impact patient safety by allowing errors to go unnoticed, limiting the ability to learn from mistakes and normalising unsafe practices.^25^ The disparities between the public and private sectors further complicate the South African health care system.^8^ In public sector settings, clinicians often face resource constraints that increase the likelihood of PSIs, while in private practice, there may be less oversight, contributing to underreporting of incidents. Incorporating practical training that prepares students for these realities is essential to minimise patient harm and ensure the anticipated clinical outcomes. The participants’ perspectives suggest a need for curricular reform to prioritise patient safety and continuing professional development to better equip clinicians for practice demands.

The findings of this study highlight the pluralistic nature of organisational support between public and private health care settings,^8,26^ such as in South Africa, where health care resources are often stretched thin, with structured reporting systems and institutional support being vital for managing PSIs. However, participants in private practice expressed frustration over the lack of standardised protocols for reporting and addressing PSIs, leaving them to navigate these incidents without formal guidance. This lack of standardised systems is a critical issue in South Africa, where the private sector plays a significant role in health care delivery but needs more regulatory frameworks in the public sector. Developing national guidelines explicitly tailored for audiology PSI reporting and management across all practice settings would improve patient safety and provide much-needed support to clinicians. In addition, it would reduce the burden on individual clinicians and promote a culture of safety and accountability across the profession. Furthermore, the implementation of the National Health Insurance (NHI) in South Africa could provide an opportunity to address these disparities. By fostering greater collaboration between the public and private sectors, NHI could help standardise practices and resource allocation, potentially reducing the fragmentation in health care systems and improving the overall management of PSIs. This would not only enhance patient safety but also strengthen regulatory frameworks and institutional support for audiologists working in diverse settings.

The direct availability of technological advancements, particularly OTC hearing aids, was highlighted as a contributor to PSIs because of the absence of professional oversight. Participants expressed concerns that unregulated access to these devices might lead to improper use and delayed professional intervention. In South Africa, where access to audiology services is already uneven, reliance on such technologies without adequate regulation could exacerbate disparities in hearing health care.^8^ While OTC devices may improve accessibility, their potential risks necessitate tighter regulatory frameworks and increased patient education. Ensuring that these technologies complement, rather than replace, professional audiological care is crucial to maintaining patient safety. As the demand for hearing health services often exceeds supply in the country,^27^ these technologies may appear to offer a solution to accessibility challenges, but the absence of stringent regulatory frameworks governing their use puts patient safety at risk. The findings call for tighter regulation to ensure that patients using OTC devices are adequately educated about their risks and benefits. In addition, continuous professional oversight should be encouraged, particularly in rural areas where audiologists may not be readily accessible.^21^

The shortage of staff and high workloads, specifically in the public sector, emerged as significant contributors to PSIs in audiology. In South Africa, audiologists face the challenge of staff shortages resource shortages and demand-supply mismatches.^27^ Participants highlighted how the pressure to manage high patient volumes often led to rushed assessments and an increased risk of errors. This study’s findings align with research conducted in Saudi Arabia, which indicated that the perceived inadequacy of staffing impacts how healthcare practitioners view and carry out their responsibilities, ultimately affecting the quality and safety of healthcare service.^28^ A study involving managers from 10 tertiary academic hospitals in South Africa concurs with the findings of this study, indicating that high turnover rates adversely affect workloads, leading to burnout, stress and dissatisfaction among staff.^29^

The participants also highlighted the delayed diagnosis and treatment of hearing loss and vestibular disorders as a result of the lack of staffing, similar to a study conducted in China.^30^ Addressing these issues requires improving staffing levels and providing audiologists with more support to manage their caseloads effectively. In addition, enhanced training on managing workloads and prioritising patient safety could help mitigate the risks associated with under-resourced environments. Systemic changes in the public and private sectors are essential to ensure that audiologists can deliver safe, high-quality care without compromising patient outcomes.

The participants emphasised the need for improved training, stricter regulation on the acquisition of new technologies and the development of clear safety protocols to reduce the incidence of PSIs. In South Africa, the regulatory environment for audiology technologies, such as OTC hearing aids, is still developing. There is also a demand for ongoing professional development to keep up with the rapid pace of technological advancement related to both testing and treatment. This is important in South Africa, where audiologists have to balance modern technology with the challenges of limited healthcare infrastructure, resources and patient education, specifically in the public sector.^21^ These recommendations align with global trends in healthcare but must be tailored to the specific socio-economic realities of South Africa. Incorporating audiology-specific patient safety measures into broader health reforms could enhance the quality of care and reduce the incidence of PSIs.

### Limitations

Although training for audiologists in South Africa is generally standardised through formal education programmes, variations in work environments and professional development opportunities may still influence how PSIs are perceived and managed. These factors may not have been fully captured in the analysis. In addition, most participants were in the early stages of their careers (ages 24 to 26 years), which may have shaped their experiences with PSIs differently compared to more experienced audiologists with greater clinical exposure.

## Conclusion

This study underscores the critical need to address PSIs in audiology through targeted instruction at the undergraduate level, followed by continuous professional development. As audiology continues to evolve with technological advancements and increasing complexities, audiologists must manage risks that impact patients’ communication abilities, balance and overall QoL. Enhancing patient safety requires improvements in training, the establishment of more effective communication strategies and stronger organisational support, with regulatory oversight essential to mitigate risks associated with emerging technologies. A comprehensive approach, encompassing curriculum reform, ongoing professional development and standardised reporting systems, is crucial for promoting a more patient-centred model of care within South African healthcare.

## Appendix 1

### Semi-structured interview guide

1. How would you define a patient safety incident in the context of audiology?
2. What do you believe are the most common types of patient safety incidents in our field?
3. Can you describe any PSIs you encountered in your practice?
4. How do PSIs impact your day-to-day clinical practice?
5. What steps do you take to prevent similar incidents in the future?
6. How adequately prepared do you feel to manage and prevent patient safety incidences?
7. What kind of training or education have you received related to patient safety? Would additional training on patient safety benefit audiologists? Elaborate
8. How do you usually become aware of PSI?
9. What is your process for reporting PSIs?
10. How do you document these incidents, and what challenges do you face?
11. How does your organization support or facilitate the reporting and management of PSIs?
12. Are there any specific policies or protocols in place at your workplace to address PSIs?
13. What improvements could be made to enhance patient safety within your organization/area of practice?
14. How do you foresee technological advancements impacting patient safety in audiology?
